# Preparedness of Lithuanian general practitioners to provide mental healthcare services: a cross-sectional survey

**DOI:** 10.1186/1752-4458-8-11

**Published:** 2014-03-24

**Authors:** Lina Jaruseviciene, Skirmante Sauliune, Gediminas Jarusevicius, Jeffrey Victor Lazarus

**Affiliations:** 1 Department of Family Medicine, Lithuanian University of Health Sciences, Kaunas, Lithuania; 2 Department of Health Management, Lithuanian University of Health Sciences, Kaunas, Lithuania; 3 Department of Cardiology, Lithuanian University of Health Sciences, Kaunas, Lithuania; 4 CHIP, Rigshospitalet, University of Copenhagen, Copenhagen, Denmark

**Keywords:** General practitioners, Teamwork, Mental healthcare, Lithuania

## Abstract

**Background:**

A large unmet need for mental healthcare in Lithuania is partially attributable to a lack of primary care providers with skills in this area. The aim of this study was to assess general practitioners’ (GPs) experience in mental healthcare and their perceptions about how to increase their involvement in the field.

**Methods:**

In this cross-sectional study, a 41-item questionnaire was distributed to a random sample of 797 Lithuanian GPs in order to investigate current practices in their provision of mental healthcare as well as their suggestions for the improvement of mental healthcare services in primary care.

**Results:**

The response rate was 52.2%. Three-quarters of the GPs agreed that they feel responsible for the management of mental health problems, but only 8.8% of them agreed that “My knowledge in mental healthcare is sufficient”. Psychiatrists were identified as the mental healthcare team specialists with whom 32% of the respondents discuss the management of their patients with a mental disorder. Collaboration with psychologists and social workers was almost threefold lower (11.6% and 12.5%). Capacity-building of GPs was found to be among the most promising initiatives to improve mental health services in primary care. Other strategies mentioned were policy level and managerial measures as well as strengthening the teamwork approach in mental healthcare.

**Conclusions:**

This study found a low self-reported competence of GPs in mental healthcare and low collaboration among GPs and other specialists in providing mental healthcare. For the situation to improve in the country, these findings point to a need for strategies to improve GPs’ expertise and teamwork in mental healthcare.

## Introduction

In many settings, the burden of mental disorders is increasing while stigma associated with these disorders coupled with insufficient specialized human resources continues. This situation calls for the development of new models for meeting mental healthcare needs [1]. Globally, the integration of mental healthcare into primary healthcare is perceived as the best way to close the treatment gap for common mental disorders [1].

The Lithuanian Mental Health Strategy (2007) has underlined the pivotal role of general practitioners (GPs) in the provision of mental healthcare [2]. However, there is an urgent need to improve mental healthcare services provided by Lithuanian GPs [3] given the state of mental health in the country. For example, the standardised suicide rate per 100 000 population for 2010 was the highest in the European Union (EU): 29.4, almost triple the E U average (10.2) [4]. Further, the registered morbidity with mental disorders is increasing (from 4,992 per 100,000 inhabitants in 2006 to 5,513 in 2012 [5]. Lithuania (like many other EU member states) does not have epidemiological data on mental illness in the general population, thus inter-country comparisons are challenging [4]. Nevertheless, antidepressant use within the last 12 month could be used as a proxy indicator of the prevalence of mental health problems in the population. This indicator in Lithuania is 11% of the general population, exceeding the EU average (7%) and the second highest in the EU [6].

Typically, primary healthcare institutions are the first point of care for people with mental health issues within the formal health system. Research reveals struggles in integrating mental health services into primary care in other countries as well. A large unmet need for mental healthcare [7] is partially attributable to a lack of primary care providers with mental healthcare skills [8]. Although GPs feel responsible for their mentally ill patients and consider themselves to be part of the care system for these patients [9], surveys performed in Australia in 1997 and 2007, for example, demonstrate that this does not necessarily enable them to meet patients’ needs: although the surveys jointly documented an overall rise in the use of mental healthcare services, the proportion of the population accessing mental healthcare from GPs remained unchanged [10].

Research performed in the Netherlands revealed similar trends: GPs’ diagnoses of mental health problems decreased and their referral to secondary care increased from 1987 to 2001 [11]. Such findings indicate a real need to build GPs’ skills in the assessment and management of mental health disorders [12] and to improve GPs’ collaboration with mental healthcare teams in general and psychiatrists in particular [13,14]. However, increased referral of mentally ill patients by GPs to secondary health care could also reflect the peculiarities of the mental healthcare sector in the country. For example, in the Netherlands the primary and secondary levels of healthcare integrate mental health services with GPs acting as gateway into secondary mental healthcare services rather than as a gatekeeper [4].

In Lithuania, primary mental health services are provided by primary healthcare teams (GPs and community nurses) as well as by mental healthcare teams (psychiatrists, psychologists and social workers). Mental healthcare teams work in mental healthcare centres, which are independent healthcare facilities that patients can access without being referred by GPs. Mental healthcare centres receive capitation fees from the national patient fund in the same way that GPs receive capitation fees for primary healthcare [15]. All primary healthcare institutions are required to have their own mental healthcare centres or to contract with other mental healthcare centres. However, research indicates a low level of collaboration between GPs with mental healthcare teams for the provision of mental healthcare services [16,17].

GPs’ actual involvement in mental healthcare provision, their competence in the field and their willingness to become more active in mental healthcare remain understudied in Lithuania. The aim of this study was to assess Lithuanian GPs’ experience in mental healthcare provision and their perceptions about how to increase their involvement in it.

## Methods

The study sample included all GPs working in Lithuania with a contract with national patient funds. Since all Lithuanian GPs who provide primary health care services have contracts with the fund, we used the complete November 2009 Lithuanian Patient Fund roster of 1800 GPs to identify a random sample of 880 GPs.

A 41-item questionnaire asked about the following:

•sociodemographic characteristics of study participants;

•self-reported experiences and attitudes regarding the provision of mental healthcare;

•reasons for referring patients to psychiatrists;

•perceptions of working environment support for mental healthcare;

•areas in which GPs would most like to increase their knowledge and skills; and

•potential measures to increase GP’s involvement in mental healthcare.

A literature review of like studies and their survey tools was carried out to inform the questionnaire together with two focus group discussions with GPs. In total, 16 GPs took part in the discussions (9 and 7). The goal was to identify the experience of GPs in mental healthcare. The focus groups discussions addressed several issues related to mental healthcare provision: collaboration practice in providing mental healthcare services, difficulties faced by GPs in providing mental health services for their patients and the potential seen by GPs to increase their involvement in mental healthcare. The thematic analysis of focus group data permitted to supplement the questionnaire with items related to collaboration practice, reasons for referral of patient to mental healthcare specialists, the perceived gaps in knowledge and skills providing mental healthcare services, and the potential measures to increase the involvement of GPs in mental healthcare. The questionnaire was pilot-tested with 18 GPs who did not take part in the focus group discussions. Minor revisions were made after pilot-testing. In 2009 the Bioethics Committee of the Lithuanian University of Health Sciences determined that its consent was not necessary for this type of study.

Questionnaires were distributed to all participants who would be at work during the 12-week survey period, which began at the end of January 2010. GPs were contacted in their primary health care centre and were informed in writing about the selection procedure, the purpose of the questionnaire and the planned publications. They were also guaranteed full confidentiality. In total, 797 questionnaires were distributed. The recipients were informed that completed questionnaires have to be enclosed in a white envelope and left in the reception of the healthcare setting. The completed questionnaires were collected one, two and three week after the distribution.

The Statistical Package for the Social Sciences for Windows version 19.0 was used to code and analyse data. Survey items intended to assess GPs’ self-reported experiences, attitudes and perceptions employed a five-point Likert scale, with responses ranging from “strongly disagree” (1 point) to “strongly agree” (5 points). The participants’ level of agreement with a series of statements relating to their involvement in the provision of mental healthcare was addressed. Answers “agree” and “strongly agree” were merged to indicate respondents’ agreement. Next, chi-square tests were used to analyse the statistical relationship between the agreement with statements and the independent variables of age (50 years old and younger and 51 years old and older), type of practice (public or private), location of practice (urban or rural) and location of mental health care centre (the same institution or different institution).

Aiming to explore the most common reasons for the patients’ referral to psychiatrists, study participants were given a list of 11 potential reasons: uncertainty of the diagnosis, uncertainty of the strategy to manage the patients’ conditions, deterioration of the status of the patient, limited possibility to prescribe psychotropic medication, lack of self-confidence in diagnosing and managing mental conditions, lack of guidelines of diagnosis and treatment of mental conditions, lack of explicitness in the roles of different healthcare professionals (e.g. GPs, psychiatrists, psychologists) in managing mental conditions, lack of support from mental healthcare teams, concern about the legal responsibility, limited time for patients consultation, and lack of financial motivation. To capture the opinion of respondents of the most promising measures to increase the involvement of GPs in mental healthcare provision, participants were given a list of 12 measures: the possibility to take part in continuing medical education events aiming to improve knowledge in mental healthcare; the possibility to take part in workshops to improve the counselling skills of mentally ill patients; the possibility to learn different psychotherapeutic methods and problems’ management techniques; greater opportunities for GPs to prescribe psychotropic medication; better description of the roles and responsibilities of GPs and mental healthcare teams; greater involvement of community nurse in mental healthcare; better GP collaboration with mental healthcare teams; the possibility to have longer consultations for mentally ill patients; better accessibility to diagnostic scales; greater attention to mental healthcare issues of the healthcare institutions’ management; the development of clear clinical guidelines for the management of mental disorders; improved possibilities for GPs to prescribe psychotropic drugs, and financial incentives for GPs involved in mental healthcare. Next, the GPs were asked to select up to three options in both situations. The distribution percentages were calculated from the total number of selected options.

## Results

### Respondents

There were 416 completed questionnaires for a response rate of 52.2%. Most respondents were female (87.3%) (Table 1). Almost one-third (30.8%) practised in private healthcare centres that had contracts with Lithuanian patient funds, and about two-thirds (67.3%) practised in public healthcare centres. A large majority of the respondents (86.1%) were in group practices rather than solo practices. Slightly less than half of the respondents (46.4%) had fewer than 1550 patients on their capitation list. Half (49.3%) indicated that a mental healthcare provider or team is situated in the same institution where they work. Additional characteristics of the respondents are presented in Table 1.

**Table 1:** Characteristics of general practitioners in the study sample

**Variable**	**Number (n = 416)**	**%**
Gender		
Male	47	11.3
Female	363	87.3
Not provided	6	1.4
Age		
≤50 years old	177	42.6
≥51 years old	234	56.2
Not provided	5	1.2
Type of practice		
Public	280	67.3
Private	128	30.8
Not provided	8	1.9
Location of practice		
Urban	294	70.7
Rural	87	20.9
Not provided	35	8.4
Size of practice		
Solo practice	31	7.5
Group practice	358	86.1
Not provided	27	6.4
Location of mental health care centre		
Same institution as general practitioner’s clinic	205	49.3
Separate institution from general practitioner’s clinic	169	40.6
Not provided	42	10.1

### GPs’ experiences and attitudes regarding the provision of mental healthcare

GPs were provided a series of statements relating to their involvement in the provision of mental healthcare (Table 2). Three-quarters of the participants (75.1%) reported agreement with the statement “I feel responsible for the management of my patients’ mental health problems”. However, their level of involvement in mental healthcare provision was lower: 53.3% of GPs indicated agreement with the statement “I often ask my patients about their mental health problems” and 56.6% with the statement “I often involve myself in the management of my patients’ mental health problems” and 81.0% with the statement “In case of suspected mental health problems, I immediately refer patients to a psychiatrist or psychologist”. GPs’ self-perceived confidence in the field of mental healthcare was even lower: 8.8% of GPs reported agreement with the statement “My knowledge in mental healthcare is sufficient.

**Table 2:** **General practitioners’ agreement**^**1 **^**with statements addressing their experiences and attitudes regarding the provision of mental healthcare services (n = 416)**

**Survey statement**	**Total sample (%)**	**Age, years old (%)**		**Type of practice (%)**		**Location of practice (%)**		**Location of MHC**^**2 **^**center**	
		**≤50**	**≥51**	**public**	**private**	**urban**	**rural**	**same institution**	**different institution**
**Current engagement in provision of mental healthcare**									
I often ask my patients about their mental health problems	53.3	**60.1**	48,1*	50.2	59.1	52.9	57.3	**48.7**	60.9*
I often involve myself in the management of my patients’ mental health problems	56.6	54.1	59.7	56.6	57.1	54.5	63.1	54.0	58.9
In case of suspected mental health problems, I immediately refer patients to a psychiatrist or psychologist	81.0	77.5	84.0	81.4	81.0	81.1	81.7	85.2	78.1
I feel responsible for the management of my patients’ mental health problems	75.1	78.6	73.2	76.8	73.1	74.0	76.3	73.2	75.6
**Self-perceived competencies**									
My knowledge of mental healthcare is sufficient	8.8	8.6	9.1	10.6	5.5	8.8	6.0	8.5	7.4
My communication skills with patients who have mental disorders are sufficient	16.8	19.4	14.4	18.6	12.6	17.8	7.3	15.5	16.5
I would be sufficiently successful at diagnosing the most common mental health disorders	20.3	21.8	18.5	20.9	19.0	19.4	21.2	17.3	22.9
**Willingness to address mental health care more at the primary healthcare level**									
I would like to be more involved in the mental healthcare of my patients	41.2	**48.5**	36.0*	43.8	36.5	**38.0**	51.8*	39.9	41.5
I would like to improve my mental healthcare knowledge and skills	86.4	**90.1**	83.1*	86.5	85.6	85.9	89.3	88.0	84.7
I would like collaborate more with mental healthcare specialists in the provision of mental health services for my patients	82.2	86.2	78.6	82.8	80.2	81.9	83.3	82.7	82.2
I think community nurses should be more involved in the provision of mental health services	75.5	75.7	75.3	**78.9**	68.3*	74.7	77.6	78.5	73.2

^1^Respondents answers “strongly agree” and “agree” were counted together.

^2^MHC center – mental healthcare center.

Values in bold indicate a statistically significant difference, * p < 0.05.

Less than half (41.2%) of the respondents agreed with the statement “I would like to be more involved in the mental healthcare of my patients”, while agreement with the statement “I would like to collaborate more with mental healthcare specialists in the provision of mental health services for my patients” was double (82.2%).

Rural GPs were more likely than urban GPs to express a willingness to be more involved in the mental healthcare of their patients (51.8% and 38%, p < 0.05). The same trend was observed among younger GPs (50 years old and younger). GPs aged 50 and younger as well as their colleagues working in primary health facilities that did not have a mental healthcare centre in the same institution more often indicated agreement with the statement “I often ask my patients about their mental health problems” p < 0.05 (Table 2).

GPs were asked to indicate their most common reasons for referring patients to psychiatrists. The highest-ranked response was uncertainty about the patient’s diagnosis (15.8%). This was followed by deterioration of the patient’s health status (13.5%); uncertainty about the strategy for managing the patient’s condition (13.1%); limited possibilities to prescribe medication (12.6%); and lack of competence in the diagnosis and treatment of mental disorders (10.6%) (data not shown in table).

### Perceptions of working environment support for mental healthcare

GPs were asked to indicate their level of agreement with a series of statements relating to working environment support for mental healthcare (Table 3). Agreement with the statement “It is possible for me to allow more time than usual for consultations with patients who have mental disorders” was reported by 11.5% of GPs, while only 5.1% of the respondents agreed with the statement “I have suitable diagnostic scales and other diagnostic instruments for the assessment of my patients’ mental health status”.

**Table 3:** **General practitioners’ agreement**^**1 **^**with statements concerning the supportiveness of working environment for mental healthcare provision (n = 416)**

**Survey statement**	**Total sample (%)**	**Age, years old (%)**		**Type of practice (%)**		**Location of practice (%)**		**Location of MHC**^**2 **^**center**	
		**≤50**	**≥51**	**Public**	**Private**	**Urban**	**Rural**	**Same institution**	**Different institution**
It is possible for me to allow more time than usual for consultations with patients who have mental disorders	11.5	9.8	13.1	10.7	12.7	10.7	12.0	11.5	10.3
I have suitable diagnostic scales and other diagnostic instruments for the assessment of my patients’ mental health status	5.1	5.2	5.1	5.7	3.9	5.4	3.2	5.4	4.9
I discuss with a psychiatrist the diagnosis, treatment and care of patients who have mental disorders	32.3	25.9	37.2	32.2	32.7	31.0	34.1	**40.0**	21.3***
I discuss with a psychologist the diagnosis, treatment and care of patients who have mental disorders	11.6	9.2	13.8	13.3	8.7	11.0	8.3	**14.4**	5.6**
A social healthcare worker takes part in providing psychosocial assistance to patients who have mental disorders	12.5	11.0	14.0	13.8	10.3	13.3	9.4	**17.2**	6.8**

^1^Respondents answers “strongly agree” and “agree” were counted together.

^2^MHC center – mental health care center.

Values in bold indicate a statistically significant difference, ** p < 0.01, ***p < 0.001.

Almost one third of the GPs (32.3%) indicated that psychiatrists are the mental healthcare team specialists with whom they discuss the management of their patients with mental disorders. Psychologists’ and social workers’ collaboration with GPs was almost threefold lower (Table 3). GPs practicing in healthcare settings that were located in the same institution as mental healthcare centres collaborated more with mental healthcare team specialists (Table 3).

### Interest in increasing knowledge and skills

Respondents were provided with a list of topics related to mental healthcare and were asked to select up to four areas in which they would like to improve their knowledge and skills (data not shown). The four highest-ranked topics were somatoform disorders, evaluation of the mental status of patients, diagnosis and management of mental disorders in advanced age, and diagnosis and management of depression (selected by 13.8%, 13.2%, 12.8% and 11.4% of the respondents, respectively).

### Measures to increase GP involvement in the provision of mental healthcare

When asked which measures would be most useful for increasing the involvement of GPs in mental healthcare, participants emphasized the necessity of building the mental healthcare capacity of GPs (possibilities to improve knowledge and skills in mental healthcare) (40.2%) (data not shown). Policy-level measures such as the development of clear clinical guidelines for the management of mental disorders, improved possibilities for GPs to prescribe psychotropic drugs, and financial incentives for GPs involved in mental healthcare were evaluated by GPs as the next highest priority (23.2%). Strengthening teamwork in mental healthcare (better description of the roles and responsibilities of GPs and mental healthcare teams, greater involvement of community nurse in mental healthcare, better GP collaboration with mental healthcare teams) and managerial measures (possibility to have longer consultations for mentally ill patients, better accessibility to diagnostic scales, greater healthcare institutions' management attention for mental healthcare issues) had almost the same importance for the GPs (18.4% and 18.2%, respectively).

## Discussion

GPs are the first health professionals encountered by many patients, it is critical for them to be able to identify and manage mental health problems. Although there have been some efforts to investigate unmet mental healthcare needs in Lithuania [3,16], this study is among the first to assess mental healthcare from the perspective of GPs: their preparedness to provide mental health services, their needs for collaboration and support as well as their ideas on how to improve the current situation.

Findings demonstrated a dramatic discrepancy between the sense of responsibility that many GPs felt about managing patients’ mental health problems and their self-perceived competencies (only 8.8% stated that their knowledge of mental healthcare is sufficient). This perceived inadequacy could be among the reasons for high patient referral rates to mental healthcare specialists. It could also be a possible explanation for the large unmet mental healthcare need in primary healthcare [3,18]. Study participants saw GP capacity-building in mental healthcare as the most promising measure to increase their involvement in the field. Studies performed in other countries also emphasise the need to improve GPs’ knowledge and skills in diagnosing and treating mental disorders [19-21]. Our study did not find differences in self-perceived competences according to the GPs’ age. This suggests that capacity building interventions should target all ages - the further education of active GPs and the curriculum of undergraduate training as well as residency in family medicine in order to ensure that graduates enter the workforce with adequate knowledge of mental illness and competence in diagnosis and treatment. Improved GPs’ knowledge and skills in mental healthcare could favourably affect the removal of restrictions to prescribe some psychotropic medications (in Lithuania there are limitations to prescribe reimbursable psychotropic drugs for the treatment of mental illnesses without prior approval of psychiatrists).

Asked to indicate which changes would be the most useful for increasing GPs’ involvement in mental healthcare provision, study participants gave the second priority to policy-level measures, including the development of clear clinical guidelines for the management of mental disorders. This is in line with study involving primary care patients in Germany, which emphasised the need to provide GPs with appropriate diagnostic categories for the mild forms of mental syndromes often seen in primary healthcare and the severe forms of comorbidity between somatoform, depressive and anxiety disorders [22]. Recently adopted Lithuanian guidelines for the assessment and management of depression and mood disorders define GPs’ duties and the boundaries of their involvement [23]. The guidance appears to be a step toward better management of mental health disorders in primary healthcare. Future research is needed, however, to assess the impact of this measure.

A large body of evidence demonstrates that the wider implementation of integrated and collaborative care models improves the management of mental health problems [24,25], improves continuity of care [26] and increases client satisfaction [27]. Collaborative care models are especially pertinent in the context of the deinstitutionalization of mental healthcare that is actively taking place in Lithuania [2]. This might open new opportunities to implement positive reforms in other parts of health system and enhance multidisciplinary collaboration. Our study revealed that GPs have low levels of collaboration with mental healthcare specialists. The most active collaboration of GPs with mental healthcare team specialists was with psychiatrists, while there was a low level of collaboration of GPs with psychologists, social workers and community nurses. Collaboration with mental healthcare team specialists was two-fold higher among GPs who practised in primary health facility with a mental healthcare centre in the same institution. This suggests that when deciding where to locate mental healthcare centres attention should be paid to their proximity to primary healthcare institutions. Moreover, specific strategies should be designed to improve collaboration of mental healthcare teams with GPs who work far from mental health care centres. One such initiative could be the regular visit of mental healthcare team members to primary healthcare settings [2].

While the proximity of mental healthcare teams to GP’s offices increases collaboration between GPs and mental health specialists, it also decreases GPs’ involvement in the routine identification of mental health problems. It might be that mental healthcare teams working in the same institution as GPs increase GPs’ reliance in the management of mental health problems of their patients without their involvement. This merits future research. A better understanding of GPs’ as well as community nurses’ and mental healthcare team specialists’ perceptions of the nature of collaboration, their experiences and expectations could be instrumental in enhancing a collaborative approach in Lithuanian primary healthcare [28].

A strengthened team approach in mental healthcare and the greater involvement of psychologists and social workers could be instrumental in addressing psychosocial aspects of mentally ill patients. Surveys indicate that GPs and nurses in other countries are also unable to manage psychosocial aspects of the care of mentally ill patients [29,30].

Our study findings are similar to findings elsewhere. For example, 57% of Norwegian GPs suggested improving collaboration with secondary mental health services personnel and 40% of them underlined the need to devote more time to patients with mental disorders in the GP context [21]. Scheduling longer time slots for patient consultations and expanding collaboration among GPs and mental healthcare specialists to encourage knowledge transfer were seen as potential strategies for improving mental healthcare by Canadian GPs as well [20].

This study has certain limitations. Firstly, the response rate was only just over 50%. However, similar peer-reviewed studies that have addressed GPs show even lower response rates [9,13,31]. Secondly, as the questionnaire was anonymous we had no possibility to check whether respondents differed substantially from non-respondents. However, the percentage of GPs practising in public health care institutions reflected the national situation: according to patient fund data 30% of Lithuanian GPs work in private primary health care centres. Moreover, the sociodemographic background of study respondents and practice characteristics were comparable with other representative studies of GPs performed in Lithuania [32,33]. Thirdly, the study had a cross-sectional design, which does not permit causal inference. Fourthly, the data collected were self-reported; triangulation including a survey among others involved in mental healthcare, and patients, would help verify them.

In spite of the aforementioned limitations, the data suggest that the problems faced by Lithuanian GPs as they try to provide healthcare services for mentally ill patients and their suggestions for how to improve the situation are in line with research findings elsewhere [20]. As it becomes more evident that different health systems face similar difficulties in integrating mental healthcare into primary care, there is an increasing need for generalizable solutions to these problems. The transferability of solutions should be assessed in future research.

## Conclusions

The readiness of GPs to provide mental healthcare services for the population is a crucial prerequisite to integrate mental healthcare services in primary healthcare. However, our study found low self-reported competences of GPs in mental healthcare and low collaboration between GPs and mental healthcare team specialists. Specific strategies should be designed by Lithuanian professional societies and medical authorities to improve the collaboration of GPs with mental healthcare specialists. GPs’ capacity building in mental healthcare was revealed as a major opportunity to increase their involvement in this regard.

## Competing interests

Authors declare no competing interests.

## Authors’ contributions

The work presented here was carried out in collaboration between all authors. LJ and JL contributed substantially to the conception and design of the study, LJ, SS and GJ contributed substantially to the collection, analysis and interpretation of the data. LJ prepared the first draft of the manuscript. LJ, SS, GJ and JL were involved in drafting the manuscript, revising it critically and providing substantial content and rewriting. All authors read and approved the final manuscript.
